# Inside the Matrix: Integrated Cytology and Molecular Testing of Thyroid FNAC Samples Using a Commercial Synthetic 3D Scaffold

**DOI:** 10.3390/ijms262211100

**Published:** 2025-11-17

**Authors:** Diana Raluca Streinu, Dana Liana Stoian, Octavian Constantin Neagoe, Mihnea Derban, Paula Diana Ciordas, Catalin Marian

**Affiliations:** 1 Department of Doctoral Studies, Victor Babes University of Medicine and Pharmacy, 300041 Timisoara, Romania; diana.streinu@umft.ro; 2 Dr. D Medical Center, 300029 Timisoara, Romania; 3 Center of Molecular Research in Nephrology and Vascular Disease, Faculty of Medicine, Victor Babes University of Medicine and Pharmacy, 300041 Timisoara, Romania; 4 2nd Department of Internal Medicine, Victor Babes University of Medicine and Pharmacy, 300041 Timisoara, Romania; 5 Second Clinic of General Surgery and Surgical Oncology, Timisoara Municipal Emergency Clinical Hospital, 300254 Timisoara, Romania; 6 Department of Pathology, CF Clinical Hospital, 300310 Timisoara, Romania; 7 Department 4—Biochemistry and Pharmacology, Victor Babes University of Medicine and Pharmacy, 300041 Timisoara, Romania; 8 Center for Complex Network Science, Victor Babes University of Medicine and Pharmacy, 300041 Timişoara, Romania

**Keywords:** CytoMatrix, thyroid cytology, fine-needle aspiration, *BRAF* V600E, molecular diagnostics, 3D-scaffold

## Abstract

Accurate preoperative assessment of thyroid nodules remains challenging, particularly in indeterminate cytological categories. Integrating molecular testing into cytology could improve diagnostic precision, enable timely intervention, and support better risk stratification and patient management. This proof-of-concept study evaluated the feasibility of performing molecular testing on fine-needle aspiration cytology (FNAC) samples processed on CytoMatrix, a three-dimensional synthetic scaffold designed to capture and preserve cellular material. Thirty-three thyroid FNAC specimens were processed on CytoMatrix, and cytological diagnoses were mirrored to the 2023 Bethesda System for Reporting Thyroid Cytopathology and correlated with final histopathology. DNA was extracted from paraffin-embedded CytoMatrix sections and analyzed for the *BRAF* V600E mutation. Adequate DNA for molecular testing was obtained in 30 of 33 cases (90%), and *BRAF* V600E mutations were detected in three papillary thyroid carcinoma samples. DNA adequacy and yield were consistent across Bethesda III–V categories, with insufficiency limited to low-cellularity Bethesda III cases. CytoMatrix enables reliable DNA recovery and targeted molecular testing without compromising cytological evaluation. This integrated cytomolecular workflow provides a feasible approach for combining cytological and molecular data in thyroid FNAC, supporting personalized and timely diagnostic management.

## 1. Introduction

Thyroid cancer represents the most common endocrine malignancy, with a steadily increasing incidence worldwide. The majority of cases are differentiated carcinomas derived from follicular cells—primarily papillary thyroid carcinoma (PTC) and follicular thyroid carcinoma—while medullary thyroid carcinoma and anaplastic thyroid carcinoma constitute rarer subtypes with distinct molecular and clinical profiles. Beyond established driver mutations such as *BRAF*, *RAS*, RET/PTC, and TERT promoter alterations, environmental and lifestyle factors, including ionizing radiation exposure, iodine imbalance, and endocrine-disrupting chemicals, may contribute to thyroid tumorigenesis by inducing oxidative stress, DNA strand breaks, and epigenetic dysregulation within follicular cells. Most differentiated thyroid carcinomas have an excellent prognosis and are highly curable when accurately diagnosed and surgically treated [1,2,3,4].

Accurate preoperative risk stratification of thyroid nodules remains a diagnostic challenge, particularly in indeterminate categories such as Bethesda III and IV, and even in some Bethesda V nodules where management uncertainty may persist, such as deciding between active surveillance and surgical intervention in nodules with borderline size or features. In current clinical practice, surgery is generally indicated for thyroid nodules larger than 4 cm, for symptomatic lesions causing compressive or aesthetic concerns, or when cytology is suspicious or malignant [5,6,7].

In recent years, ultrasound-based risk stratification systems such as the American College of Radiology Thyroid Imaging Reporting and Data System (ACR TI-RADS) and the European Thyroid Association TIRADS (EU-TIRADS) have become integral to the diagnostic workup of thyroid nodules. These standardized scoring systems provide objective criteria to estimate malignancy risk based on sonographic features, thereby guiding the indication for FNAC and subsequent management. Combining ultrasound risk categories with cytological findings has been shown to enhance preoperative risk assessment, particularly in indeterminate nodules, supporting more individualized and evidence-based clinical decision-making [8].

Although ancillary molecular testing has emerged as a valuable adjunct for enhancing diagnostic certainty and guiding clinical management of thyroid nodules, its use remains limited in routine cytology practice due to sample-related and logistical constraints [9,10]. Conventional cytological smears, while essential for morphologic assessment, are generally less suited for molecular testing due to insufficient cellular material, fixation-related degradation, and the absence of residual sample for extraction [11,12,13]. In current cytology practice, such testing is typically performed on cell block preparations. However, traditional cell block techniques are often restricted by inconsistent cellular yield, variable processing quality, and susceptibility to technical artifacts. Moreover, their preparation requires specialized equipment, trained personnel, and multiple processing steps, making them both resource-intensive and time-consuming in routine diagnostic settings [14,15].

Genetic alterations play a critical role in the biology and clinical behavior of thyroid cancer. Among these, *BRAF* V600E, *RAS* mutations, rearrangements such as RET/PTC and PAX8/PPARγ, and alterations in the TERT promoter are the most extensively studied, given their relevance for diagnosis, prognosis, and risk stratification [16,17,18]. Reliable detection of such markers, however, requires high-quality, well-preserved cytological material—conditions that are not consistently achieved with traditional FNAC smears [19].

Recent advances in synthetic support matrices have opened new possibilities for cytological sample preservation and processing. CytoMatrix, a novel three-dimensional commercial synthetic scaffold, allows for efficient entrapment and preservation of cells from FNAC specimens, creating a paraffin-embedded format more compatible with downstream molecular techniques. Unlike traditional smear preparations, CytoMatrix preserves cellular architecture and yields sufficient material for both cytological evaluation and nucleic acid extraction, thereby enabling a dual-purpose diagnostic pathway. This technology represents a practical solution for integrating molecular diagnostics into routine cytopathology without altering existing clinical sampling procedures [20,21,22].

In this study, we investigated the feasibility and reliability of performing molecular testing on CytoMatrix-processed thyroid FNAC samples. *BRAF* V600E analysis was selected as a proof-of-concept marker because of its well-established role in papillary thyroid carcinoma, the most common form of thyroid malignancy [23,24,25]. The primary aim, however, was not to quantify *BRAF* V600E prevalence but to validate the molecular compatibility of CytoMatrix and to assess whether an integrated algorithm—FNAC followed by CytoMatrix processing and subsequent mutation analysis—can serve as a robust, clinically applicable workflow for thyroid nodule evaluation.

In this context, the present study explores the potential of CytoMatrix as a practical platform for routine, integrated cytology and molecular workflows, addressing limitations of conventional smears and cell blocks.

By enhancing the molecular diagnostic yield of FNAC specimens, this approach may help avoid unnecessary thyroidectomies in patients with indeterminate nodules harboring low-risk molecular profiles, while supporting timely and appropriate surgical intervention in cases exhibiting molecular alterations associated with malignancy or aggressive behavior [26,27]. While *BRAF* V600E was selected in this study as a proof-of-concept marker due to its high specificity for papillary thyroid carcinoma, CytoMatrix has the potential to support more comprehensive molecular testing strategies beyond single-gene analysis [20,28,29]. In this context, CytoMatrix serves not merely as a preservation scaffold but as a critical enabler of a practical, precision-oriented diagnostic workflow that integrates cytology with molecular analysis. Its implementation in routine practice holds promise for advancing individualized, risk-adapted management of thyroid nodular disease.

This study aimed to evaluate the use of CytoMatrix for molecular applications by demonstrating that DNA extracted from CytoMatrix material is suitable for downstream testing. Beyond establishing DNA adequacy, our goal was to confirm that molecular analyses can be reliably performed using *BRAF* V600E as a validation marker. Importantly, this study was conceived as a technical proof-of-concept—to evaluate whether CytoMatrix-processed FNAC material can sustain integrated cytological and molecular workflows, providing a foundation for larger multicenter validation studies.

## 2. Results

### 2.1. Study Population

33 patients were included, comprising 26 females (78.8%) and 7 males (21.2%). The age of participants ranged from 23 to 74 years.

DNA was successfully extracted from the vast majority ofFormalin-Fixed Paraffin-Embedded (FFPE) CytoMatrix samples. The mean concentration was 5.68 ± 3.48 ng/μL, with values ranging from 1.6 to 13.7 ng/μL. Using a threshold of 3 ng/μL as the minimum adequacy requirement for molecular testing, 30/33 samples (90%) were suitable for analysis. Three cases, all classified cytologically as Bethesda III, failed to yield sufficient DNA. Of these, two were benign and one malignant on final histopathology. Overall DNA adequacy was high, with insufficiency limited to three low-cellularity cases, all within the Bethesda III category.

It should be noted that DNA adequacy refers to the total extracted DNA, which may contain both tumoral and non-tumoral components. To minimize this, all CytoMatrix blocks were reviewed by a pathologist to ensure sufficient cellularity before extraction, thereby enriching tumor content. In addition, the ONCO-*BRAF* assay (Generi-Biotech, Hradec Králové, Czech Republic) demonstrates a high analytical sensitivity, with a limit of detection as low as 0.05% mutant *BRAF* alleles within a wild-type background at 100,000 DNA copies per reaction as reported by the manufacturer (Generi-Biotech, 2024) [30]. This high sensitivity minimizes the influence of background non-tumoral DNA and supports reliable detection of low-abundance *BRAF* V600E mutations in FNAC-derived samples [31,32].

Baseline demographic and molecular characteristics are summarized in Table 1.

### 2.2. Histopathology Outcomes

All 30 nodules with adequate DNA had available histopathological diagnoses, as only cases that had undergone thyroidectomy were included for molecular analysis; the histopathological diagnoses were evaluated according to the 2022 World Health Organization (WHO) Classification of Endocrine and Neuroendocrine Tumors. Based on these histopathological results, 17 nodules (56.7%) were malignant and 13 (43.3%) were benign. The malignant group consisted mainly of papillary thyroid carcinoma (PTC, 10 cases, 33.3%) and its follicular variant (FV-PTC, 6 cases, 20.0%). The benign group was dominated by nodular goiter (11 cases, 36.7%). In addition, two follicular adenomas (6.7%) and one medullary carcinoma (3.3%) were identified [33].

Table 2 presents the detailed histopathological distribution of the study group (30 cases).

### 2.3. BRAF V600E Results

*BRAF* V600E mutations were detected in 3/30 patients (10.0%). All mutation-positive cases were classic PTC on final histology and cytologically Bethesda V. No mutations were found in FV-PTC, follicular adenomas, medullary carcinoma, or benign nodular goiter.

This distribution highlights the high specificity of *BRAF* V600E for PTC, but also illustrates its limited sensitivity, since most PTC cases in this cohort were mutation-negative. Fisher’s exact test confirmed the association between *BRAF* positivity and malignancy (*p* = 0.030).

Molecular analysis was completed in all 30 cases with adequate DNA, regardless of mutation status, demonstrating that testing could be performed in nearly all samples.

Table 3 summarizes the distribution of *BRAF* mutations by histopathology.

### 2.4. Bethesda Category Distribution and Patterns

Preoperative cytology in the 30 cases with adequate DNA was classified as Bethesda III in 14 cases (46.7%), Bethesda IV in 2 cases (6.7%), and Bethesda V in 14 cases (46.7%).

As expected, the likelihood of malignancy increased with higher Bethesda categories. Malignancy rates were 21.4% in Bethesda III, 0% in Bethesda IV, and 100% in Bethesda V. Chi-square testing confirmed a strong association between Bethesda category and malignancy (χ^2^ = 20.40, df = 2, *p* < 0.001; Cramér’s V = 0.83).

All *BRAF* mutations were confined to Bethesda V nodules (3/14, 21.4%). No mutations were observed in Bethesda III or IV. When analyzed excluding the three Bethesda III cases with insufficient DNA, the distribution remained the same: 0/11 positive in Bethesda III, 0/2 positive in Bethesda IV, and 3/14 positive in Bethesda V. Fisher’s exact test on adequate samples showed a trend toward association (*p* = 0.093), though this did not reach statistical significance due to the limited number of mutation-positive cases.

**Figure 1:**
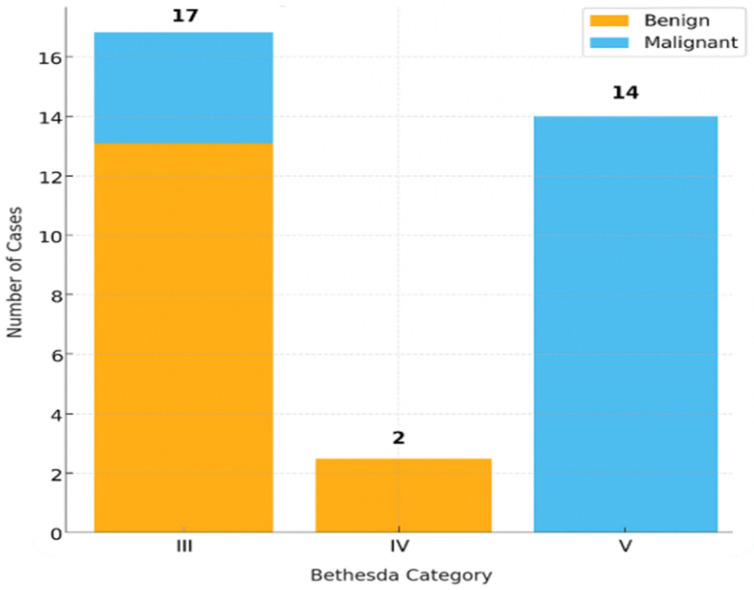
Distribution of final histopathological diagnoses (benign vs. malignant) according to Bethesda categories III–V total 33.

**Figure 2:**
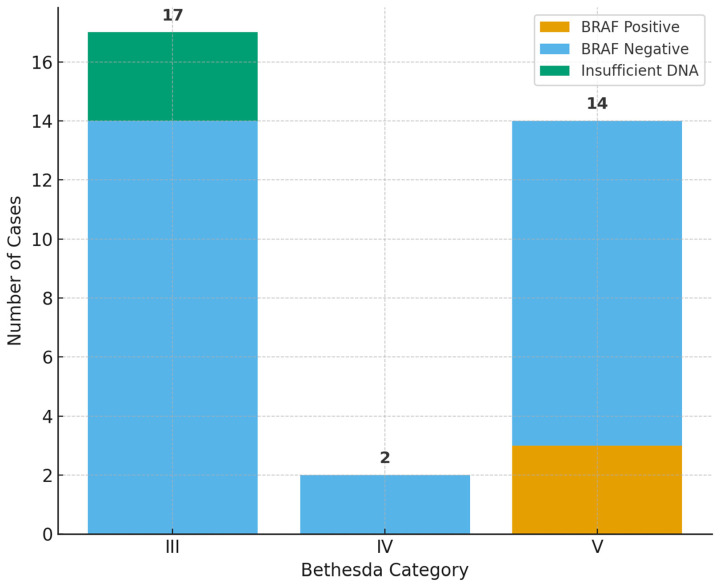
Distribution of *BRAF* V600E mutation status across Bethesda categories.

The correlation between Bethesda category and histopathology is presented in Table 4 and Figure 1, and the distribution of *BRAF* status across Bethesda categories is shown in Table 5 and Figure 2.

### 2.5. DNA Quantity in Relation to Outcomes

The mean DNA concentration in *BRAF*-positive cases was 4.07 ng/μL, compared with 5.86 ng/μL in *BRAF*-negative cases (Mann–Whitney U = 31.5, *p* = 0.556). Malignant samples yielded 6.51 ng/μL on average, compared with 4.61 ng/μL for benign samples (Mann–Whitney U = 139.5, *p* = 0.232).

When analyzed by Bethesda category, median DNA concentration did not differ significantly (Kruskal–Wallis H = 3.96, *p* = 0.138). Pairwise Mann–Whitney tests with Bonferroni correction confirmed the absence of significant differences between categories. DNA adequacy was consistent across diagnostic categories, with insufficiency limited to three Bethesda III cases.

## 3. Discussion

The present study provides preliminary support that CytoMatrix can feasibly enable an integrated diagnostic workflow, in which both cytological and molecular analyses are performed from the same FNAC material. This dual-purpose capability represents a crucial step toward practical implementation of personalized diagnostics within routine cytology. While *BRAF* V600E was selected as a proof-of-concept marker because of its established role in papillary thyroid carcinoma, the broader implication lies in showing that CytoMatrix may serve as a platform for routine molecular testing within standard cytopathology practice [25,34]. By enabling DNA extraction alongside cytomorphological assessment, this approach suggests a pathway toward more integrated and precision-oriented diagnostics for thyroid nodules.

### 3.1. CytoMatrix as a Technical Solution to Cytology Limitations

One of the persistent challenges in thyroid cytology is the limited cellularity of FNAC samples, which can restrict ancillary studies such as immunocytochemistry or molecular testing. Conventional smears often exhaust the material available, and cell-block techniques, while valuable, can be inconsistent in quality and sometimes yield insufficient DNA for reliable testing [15,35,36]. CytoMatrix, a tridimensional scaffold designed to capture and preserve cellular material, addresses this limitation by standardizing sample collection and ensuring that nucleic acids can be extracted for further analysis [20,22].

In our cohort, DNA concentrations obtained from CytoMatrix were consistently sufficient for mutation testing, with a mean yield of 5.68 ng/μL. Overall, 90% of samples exceeded the adequacy threshold of 3 ng/μL, confirming the robustness of the platform. The three inadequate cases all belonged to Bethesda III, of which two were benign and one malignant on final histopathology. These findings suggest that inadequacy was more likely related to technical and sampling limitations rather than to the biological nature of the lesion. No inadequate samples were observed in Bethesda IV or V. It should also be noted that DNA insufficiency at the level of a single CytoMatrix slice does not necessarily imply that the entire block is unsuitable, as other sections might still provide sufficient material. The observation that one malignant case failed to yield adequate DNA further indicates that inadequacy should not be interpreted as reassuring, but rather as a technical limitation that requires cautious clinical consideration.

### 3.2. BRAF V600E as a Proof of the Concept

To illustrate the molecular testing potential of CytoMatrix, we performed targeted analysis for the *BRAF* V600E mutation, the most common genetic alteration in papillary thyroid carcinoma [25,34]. In our series, three patients tested positive, all of whom had previously confirmed classic PTC on histopathology and Bethesda V cytology. No *BRAF* mutations were detected in benign nodular goiter, follicular adenomas, FV-PTC, or medullary carcinoma. These findings are consistent with the established biology of thyroid tumors: *BRAF* mutations are strongly associated with classic PTC, while FV-PTC is more frequently driven by *RAS* mutations, and medullary carcinoma by *RET* alterations [37,38,39].

Although the absolute number of mutation-positive cases was small, the distribution of *BRAF* positivity exclusively within Bethesda V nodules reinforces two key points. First, the CytoMatrix platform is technically capable of supporting molecular analysis, as demonstrated by reliable mutation detection in cases where it is biologically expected. Second, the fact that several Bethesda V nodules were *BRAF*-negative underscores the limited sensitivity of single-gene testing, even in highly suspicious categories. Likewise, all indeterminate nodules (Bethesda III and IV) in our series were *BRAF*-negative, despite some proving malignant on histopathology. These findings highlight that while *BRAF* testing can provide strong confirmatory evidence when positive, its absence does not rule out malignancy. This further supports the need for multigene panels that include additional drivers such as *RAS*, RET/PTC, and TERT to improve risk stratification across both indeterminate and suspicious nodules [40,41,42,43,44]. Therefore, while single-gene analysis may offer limited diagnostic information, the primary objective here was to validate the molecular compatibility of the CytoMatrix platform. The successful amplification and detection of *BRAF* V600E mutations in appropriate cytological contexts confirm that the scaffold preserves nucleic acid integrity suitable for downstream analysis.

DNA extraction was successful in 30 out of 33 nodules (90%), confirming the overall robustness of CytoMatrix for molecular testing. Within the indeterminate Bethesda III category, 14 of 17 cases yielded sufficient DNA, while three samples fell below the adequacy threshold (two benign and one malignant). Although all Bethesda III cases were *BRAF*-negative, this was consistent with expectations, since only a minority were malignant. These results show that CytoMatrix ensures adequate DNA recovery across most indeterminate lesions, thereby providing a reliable substrate for molecular testing. While single-gene analysis offered limited diagnostic contribution in this cohort, the ability to consistently extract DNA supports the primary objective of this study, namely, to explore whether molecular testing can be applied to CytoMatrix-derived material. *BRAF* V600E was selected as a proof-of-concept marker, but the platform may also be suitable for multigene panels that could provide greater diagnostic value, particularly in indeterminate categories where single-gene testing is often insufficient [20,41,42].

Molecular testing of FNAC material may be influenced by the presence of non-tumoral DNA and by fixation-related damage that compromises template integrity. Formalin-induced cross-linking, base modifications, and nucleic-acid fragmentation can interfere with polymerase activity, reducing amplification efficiency and, in some cases, producing invalid results despite apparently adequate DNA yield. Prolonged fixation duration and subsequent processing steps—such as dehydration, embedding, or storage—can further intensify DNA fragmentation and cross-linking, increasing the risk of amplification failure. Additionally, DNA degradation or chemical alteration may occur during extraction, particularly in samples with suboptimal preservation. These considerations highlight the critical importance of pre-analytical quality control, including standardized fixation, controlled processing times, and the selection of well-preserved, representative CytoMatrix sections for molecular analysis [45,46,47,48].

### 3.3. Comparison with Previous Literature

The prevalence of *BRAF* mutations in PTC varies widely across studies, typically ranging between 40% and 80%, with geographic and histological influences [49,50,51]. The lower prevalence (10%) is explained not only by the small sample size but also by the composition of our cohort, which included benign nodules and Bethesda III cases in addition to malignant lesions. As these categories are not expected to harbor *BRAF* mutations, their inclusion diluted the apparent mutation frequency. This emphasizes that the present study should be interpreted within the context of technical feasibility rather than epidemiological prevalence, as the cohort composition and scope were optimized for workflow validation. Similar findings have been reported in other single-institution cohorts, where *BRAF* testing demonstrated high specificity but lower sensitivity for thyroid cancer detection [52,53].

### 3.4. Clinical Implications

The ability to perform both cytology and molecular testing on a single FNAC sample has direct implications for patient care. In current practice, indeterminate cytology often leads to repeat aspirations or diagnostic surgery, which may ultimately prove unnecessary. By incorporating molecular testing into the initial workup, clinicians can refine risk estimates and tailor management strategies more appropriately [54,55].

Molecular testing also has the potential to directly influence clinical decision-making. In indeterminate categories (Bethesda III and IV), where morphology alone cannot reliably distinguish benign from malignant lesions, molecular results could help clinicians avoid unnecessary diagnostic surgeries in mutation-negative cases, while guiding timely intervention when mutations are present [56,57]. In suspicious nodules (Bethesda V), molecular testing may refine surgical planning, either by preventing overly extensive procedures in mutation-negative cases or by ensuring appropriate definitive surgery when high-risk mutations are detected [58,59]. This approach aligns with current international recommendations, including the 2019 ESMO Clinical Practice Guidelines, which advocate a risk-adapted strategy for the management of thyroid nodules and cancers. By integrating molecular data into the diagnostic pathway, clinicians can more effectively balance the risks of overtreatment against the need for timely and adequate intervention [60,61].

In our series, although *BRAF* testing itself did not change the interpretation of Bethesda III and IV nodules, the successful application of molecular analysis on CytoMatrix-derived material suggests that the platform may accommodate broader gene panels. This could be particularly relevant for Bethesda III, where only a small proportion of nodules proved malignant and none harbored *BRAF* mutations. In such cases, extended panels including *RAS*, RET/PTC, and TERT mutations may provide greater diagnostic yield.

Another important implication is workflow efficiency. CytoMatrix allows for simultaneous morphological and molecular assessment without requiring separate aspirations or additional sample preparations. This reduces patient discomfort, minimizes procedure time, and standardizes sample processing. The method also facilitates archiving, as the remaining matrix can be stored and revisited for further testing if new biomarkers emerge [20,62].

CytoMatrix fulfills all the necessary technical and diagnostic criteria to serve as a stand-alone platform, providing adequate material for both cytological and molecular evaluation. Nevertheless, based on our study results and practical experience, we recommend that during the initial stages of implementation, CytoMatrix be used as a complementary tool alongside conventional smears, particularly in diagnostic laboratories and clinical centers employing it as a cell-scaffold system. This phased approach allows clinicians and laboratory teams to adapt to the workflow and ensures diagnostic consistency as standardized protocols are gradually established. Even when used as a complementary tool, CytoMatrix holds substantial value by improving sample management, analytical efficiency, and integration between cytological and molecular data.

From an operational perspective, CytoMatrix can be seamlessly integrated into standard cytopathology workflows, as it employs routine fixation and embedding techniques and does not require specialized instrumentation. This procedural compatibility supports broad clinical adoption and minimizes implementation barriers [20].

Furthermore, by enabling concurrent cytological and molecular analyses from a single FNAC sample, CytoMatrix may offer cost advantages through improved sample efficiency, fewer repeat procedures, and better preoperative risk stratification, ultimately contributing to more rational use of healthcare resources.

### 3.5. Integration with Bethesda System

Our results confirmed the expected stepwise increase in malignancy rates from Bethesda III to V, thereby validating the internal consistency of our cohort. Malignancy rates rose from 21.4% in Bethesda III to 100% in Bethesda V (14/14; 95% CI 77–100%), with a statistically significant overall association (χ^2^ = 20.401, df = 2, *p* < 0.001). The effect size was very strong (Cramér’s V = 0.825), supporting the robustness of the cytological classification within this series. It is important to note that CytoMatrix is not yet standardized within the official Bethesda system; in this study, categories were applied in a mirrored, reference manner to align as closely as possible with the 2023 Bethesda criteria, rather than as formal Bethesda diagnoses [63].

Regarding molecular findings, *BRAF* V600E mutations were detected exclusively in Bethesda V nodules (3/14, 21.4%). When cases with inadequate DNA were excluded, Fisher’s exact test demonstrated a tendency toward association between *BRAF* positivity and Bethesda category (*p* = 0.093). Although this did not reach statistical significance—most likely due to the small number of mutation-positive cases—the distribution is consistent with the established biology of papillary thyroid carcinoma, in which *BRAF* mutations predominantly occur in morphologically suspicious nodules [64,65].

Taken together, these findings indicate that CytoMatrix-derived material was adequate not only for cytological classification but also for reliable downstream molecular testing. The 100% malignancy rate in Bethesda V should be interpreted cautiously, as it likely reflects enrichment for operated nodules and the relatively small sample size. In addition, in our institution CytoMatrix FNAC was preferentially applied to nodules with suspicious ultrasound features, which may have further contributed to the elevated malignancy rate in Bethesda V. Nonetheless, it is also conceivable that CytoMatrix contributed to improved cytomorphological assessment, thereby reducing false-positive Bethesda V classifications. Larger, multicenter studies will be required to determine whether CytoMatrix enhances the predictive accuracy of indeterminate and suspicious cytology categories.

### 3.6. Strengths and Limitations

This study was intentionally designed as a feasibility and validation investigation rather than a powered diagnostic trial. Accordingly, the focus was on verifying DNA recovery and molecular compatibility rather than estimating population-level mutation rates.

The strengths of our study include the practical application of CytoMatrix, the systematic correlation of cytology, histopathology, and molecular testing, and the demonstration of consistent DNA recovery across diagnostic groups. The integration of statistical analyses further reinforces the robustness of our observations.

Limitations should also be acknowledged. The study was conducted in a single center with a relatively small sample size, which restricts the generalizability of prevalence estimates.

Only one genetic marker (*BRAF* V600E) was analyzed, selected as a proof of concept due to its clinical relevance in PTC. As expected with this focused approach and smaller sample size, the number of mutation-positive cases was limited, which widened the confidence intervals of statistical estimates. The absence of mutations in Bethesda III and IV nodules should also be interpreted in context: our cohort included a predominance of benign lesions, and the analysis was limited to a single marker. More importantly, the scope of this study was not to characterize the full mutational spectrum, but to determine whether molecular testing can be reliably performed on CytoMatrix material. Importantly, DNA could be successfully extracted across diagnostic categories, including most indeterminate lesions, with insufficiency occurring only in a minority of Bethesda III cases.

DNA was extracted from only one 10-µm section per CytoMatrix block, which proved sufficient for targeted testing in most cases. Additional sections could be processed to increase DNA recovery, a strategy that may be particularly important when applying broader molecular panels or next-generation sequencing, where higher input requirements are anticipated [66,67]. Another limitation is that DNA extraction kits recover total DNA without distinguishing tumoral from non-tumoral components [32]. Although pathologist review was used to enrich tumor content, some background DNA is unavoidable and may influence molecular results. However, as mentioned, the analytical sensitivity of real-time PCR assays such as the ONCO-*BRAF* kit—capable of detecting at frequencies as low as 0.05% mutant *BRAF* alleles within a wild-type background at 100,000 DNA copies per reaction—helps minimize the impact of background non-tumoral DNA on mutation detection [30].

In addition to the limited sample size, ultrasound and TI-RADS classification data were not systematically recorded and could not be analyzed in correlation with cytological or molecular findings. Moreover, inter-observer reproducibility for cytological assessment on CytoMatrix preparations was not evaluated, which may affect the generalizability of diagnostic performance across observers. Future studies should aim to include standardized ultrasound documentation and multi-observer evaluation to strengthen external validation.

Larger, multicenter studies applying comprehensive mutation panels will be required to capture the full genetic heterogeneity of thyroid nodules and to refine risk stratification, particularly in indeterminate categories. Despite these limitations, a notable strength of this study is that molecular testing proved technically feasible in nearly all samples, supporting the potential practical applicability of CytoMatrix in real-world cytology practice.

### 3.7. Future Perspectives

CytoMatrix may provide a technical foundation for moving beyond single-marker assays. By facilitating adequate DNA recovery, the platform could enable the application of extended assays, including next-generation sequencing (NGS) panels covering multiple driver mutations and rearrangements. In this way, CytoMatrix has the potential to help bridge the gap between traditional cytology and precision molecular diagnostics.

Recent evidence has shown that CytoMatrix-processed samples can successfully undergo multigene molecular analysis, demonstrating its compatibility with broader NGS panels and confirming its suitability for detecting multiple clinically relevant alterations such as *BRAF*, *RAS*, RET/PTC, and TERT [22]. Building on these encouraging results, future research should continue to explore and expand multigene testing using CytoMatrix-derived material to validate its performance across a wider range of molecular targets and clinical contexts.

Such studies may help clarify the role of CytoMatrix in refining the management of Bethesda III and IV nodules, where single-marker testing remains insufficient. Prospective, multicenter cohorts would also be valuable to confirm reproducibility and to establish standardized workflows. In addition, cost-effectiveness analyses are warranted to determine whether integrating CytoMatrix with molecular testing can reduce unnecessary surgeries and improve patient management in routine clinical practice [43,68].

## 4. Materials and Methods

### 4.1. Study Design and Population

This prospective, single-center study was conducted at Dr. D Medical Center in Timișoara, Romania, between June 2024 and June 2025. Patients undergoing FNAC for the evaluation of thyroid nodules were enrolled based on predefined eligibility criteria.

### 4.2. Inclusion and Exclusion Criteria

The study included patients with suspicious thyroid nodules identified on ultrasound who underwent FNAC, with the aspirated material processed using the CytoMatrix synthetic scaffold. Cytological diagnoses were assigned according to the Bethesda System (categories III—Atypia of Undetermined Significance, IV—Follicular Neoplasm, or V—Suspicious for Malignancy), as adapted for CytoMatrix-derived material [63]. Only cases that subsequently underwent thyroidectomy and had an available final histopathological diagnosis were included in the molecular analysis. Additional inclusion criteria were the availability of adequate cytological material for diagnosis and provision of informed consent.

Exclusion criteria were non-diagnostic cytology (Bethesda-referenced I), clearly benign cytology (Bethesda-referenced II), as well as patients with a known history of thyroid carcinoma or without histopathological follow-up.

**Figure 3:**
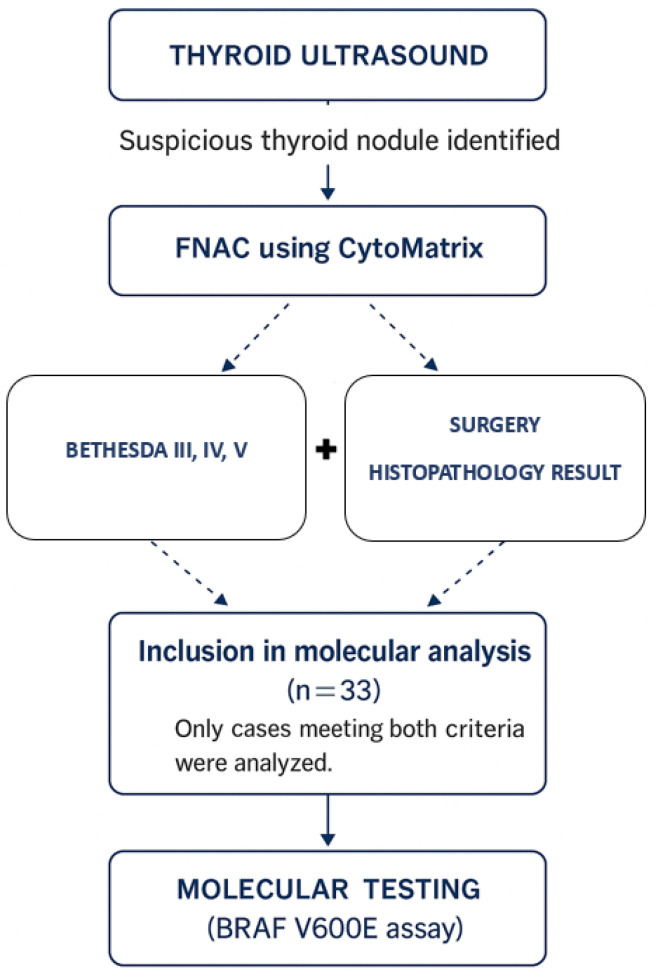
Flowchart illustrating patient selection and inclusion criteria for the study. After thyroid ultrasound and FNAC using CytoMatrix, cases classified as Bethesda III, IV, or V and with available histopathological confirmation following surgery were included in the molecular analysis (*n* = 33).

The inclusion workflow and selection criteria for molecular analysis are illustrated in Figure 3.

### 4.3. Sample Processing and Cytological Classification

**Figure 4:**
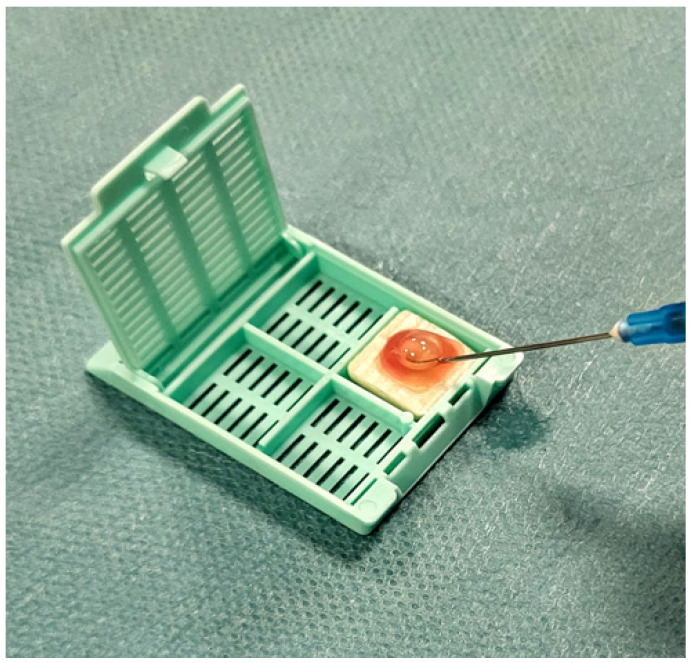
Deposition of fine-needle aspiration material onto the CytoMatrix scaffold.

Ultrasound-guided FNAC was performed using a single needle pass with a 23–25 gauge needle attached to a 10 mL syringe, employing both capillary and aspiration techniques to optimize cellular yield [69,70]. The aspirated material was immediately deposited onto the CytoMatrix synthetic scaffold (developed byUCS Diagnostics Srl & Campus Bio-Medico, University of Rome, Rome, Italy), as shown in Figure 4.

The scaffold was placed into a cassette and fixed in 10% neutral buffered formalin for at least 12 h. Following fixation, CytoMatrix scaffolds were processed according to routine pathology protocols, embedded in paraffin, and sectioned into 4 -µm slices. Initial sections were used for cytological evaluation, after which each block was reviewed by a pathologist to assess cellularity. Based on this review, additional representative 10-µm sections (one from each CytoMatrix block) were selected and cut for DNA extraction, thereby reducing the risk of sampling predominantly cystic or non-neoplastic material and increasing the likelihood of obtaining diagnostically relevant DNA.

**Figure 5:**
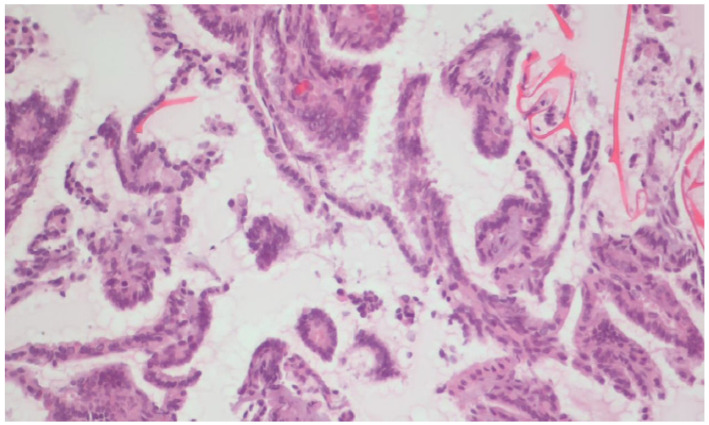
Cytomorphological appearance of a CytoMatrix section after routine H&E staining, showing strong features of papillary thyroid carcinoma, 20× magnification.

Representative cytological morphology obtained from CytoMatrix sections is shown in Figure 5, demonstrating preservation of cellular details suitable for diagnosis in parallel with molecular testing.

Cytological diagnoses were assigned on CytoMatrix-derived sections with reference to the 2023 Bethesda System, applying its criteria as closely as possible in this non-standardized medium. Throughout this study, the terms Bethesda III–V in this manuscript refer to Bethesda-referenced categories applied on CytoMatrix material. Only nodules classified as Bethesda categories III, IV, or V were included, as these represent indeterminate or suspicious lesions where ancillary molecular testing is most relevant [56,58].

### 4.4. DNA Extraction and Molecular Testing

Genomic DNA was extracted from CytoMatrix-derived formalin-fixed paraffin-embedded (FFPE) blocks using the QIAamp DNA FFPE Advanced Kit (Qiagen, Hilden, Germany), according to the manufacturer’s protocol [71]. Detection of the *BRAF* V600E mutation was performed with the ONCO-*BRAF* (V600E) PCR Kit (Generi Biotech, Hradec Králové, Czech Republic), a qualitative real-time PCR assay designed to identify the V600E substitution at codon 600 of the *BRAF* gene [30]. All molecular analyses were carried out in the Department of Biochemistry, University of Medicine and Pharmacy “Victor Babeș”, Timișoara, Romania, which provides molecular testing and research facilities.

We selected *BRAF* V600E as the molecular target in this study both to evaluate the feasibility of incorporating mutation testing into the workflow of indeterminate cytology cases and because it represents the most frequent and clinically relevant genetic alteration in papillary thyroid carcinoma [64,65].

## 5. Conclusions

This preliminary study suggests that CytoMatrix may serve as a viable platform for integrated cytological and molecular testing of thyroid nodules. Adequate DNA yields were obtained in 90% of cases, supporting the application of mutation analysis. While *BRAF* V600E was chosen as an illustrative marker, the broader implication is that CytoMatrix could support a wider range of molecular assays, with potential relevance for precision diagnostics in thyroid cytology. Given these encouraging results, future studies should expand molecular analysis of CytoMatrix-derived material to include broader mutation panels, thereby enhancing the clinical utility of this integrated diagnostic approach. By enabling simultaneous morphological and genetic characterization from a single FNAC, CytoMatrix may contribute to improving diagnostic accuracy, reducing the need for repeat procedures, and supporting more individualized patient care. Future multicenter studies with expanded molecular panels are warranted to confirm reproducibility and to establish standardized CytoMatrix-based workflows for integrated cytomolecular diagnostics.

## Figures and Tables

**Table 1:** Baseline demographic and clinical characteristics of the study population. The table summarizes patient age, sex distribution, and relevant clinical variables for the 33 individuals who underwent thyroid FNAC and CytoMatrix processing.

Variable	Result
Age	23–74 years
Sex	26 F (78.8%), 7 M (21.2%)
DNA Concentration (ng/μL)	5.68 ± 3.48 (range 1.6–13.7)
Sufficient DNA	30/33 (90%)
*BRAF* V600E-Positive	3
*BRAF* V600E-Negative	27

**Table 2:** Final histopathological diagnoses of the 30 thyroid nodules analyzed. Histopathological outcomes were derived from surgical resection specimens and used as the reference standard for diagnostic correlation with cytological categories (Bethesda III–V).

Diagnosis	n	%
Benign Nodular Goiter	11	36.7
Papillary Thyroid Carcinoma	10	33.3
Follicular Variant of PTC (FV-PTC)	6	20.0
Follicular Adenoma	2	6.7
Medullary Carcinoma	1	3.3

**Table 3:** *BRAF* V600E mutation status in relation to final histopathological diagnosis of thyroid nodules. The table summarizes mutation detection results across histological outcomes identified in the study cohort.

Mutation	Malignant	Benign	Total
*BRAF*-Positive	3	0	3
*BRAF*-Negative	14	13	27
Total	17	13	30

**Table 4:** Comparison of Bethesda cytology categories with corresponding final histopathological diagnoses in the study cohort (*n* = 33). The table illustrates the distribution of benign and malignant outcomes within each cytological category to assess diagnostic concordance.

Bethesda	Malignant	Benign	Total
III	4	13	17
IV	0	2	2
V	14	0	14

**Table 5:** Distribution of *BRAF* V600E mutation status across Bethesda cytology categories III–V in 33 thyroid nodules. The table illustrates the relationship between cytological classification and molecular findings, showing the frequency of *BRAF*-positive and *BRAF*-negative cases within each category.

Bethesda	*BRAF*-Positive	*BRAF*-Negative	Insufficient DNA	Total
III	0	14	3	17
IV	0	2	0	2
V	3	11	0	14

## Data Availability

The datasets supporting the conclusions of this article are included within the article. Further inquiries can be directed to the corresponding author.

## Abbreviations

The following abbreviations are used in this manuscript:

EU-TIRADS	European Thyroid Imaging Reporting and Data System
DNA	Deoxyribonucleic Acid
FNAC	Fine-Needle Aspiration Cytology
PCR	Polymerase Chain Reaction
FFPE	Formalin-Fixed Paraffin-Embedded
PTC	Papillary Thyroid Carcinoma
FVPTC	Follicular Variant of Papillary Thyroid Carcinoma
ESMO	European Society for Medical Oncology
WHO	World Health Organization
